# Assessing the In Vitro Activity of Selected Porphyrins in Human Colorectal Cancer Cells

**DOI:** 10.3390/molecules27062006

**Published:** 2022-03-21

**Authors:** Maciej Piotr Frant, Mariusz Trytek, Roman Paduch

**Affiliations:** 1 Department of Swine Diseases, National Veterinary Research Institute, Al. Partyzantów 57, 24-100 Puławy, Poland; maciej.frant@piwet.pulawy.pl; 2 Department of Industrial and Environmental Microbiology, Institute of Biological Sciences, Maria Curie-Skłodowska University, 20-033 Lublin, Poland; mariusz.trytek@mail.umcs.pl; 3 Department of Virology and Immunology, Institute of Biological Sciences, Maria Curie-Skłodowska University, Akademicka 19, 20-033 Lublin, Poland; 4 Department of General and Pediatric Ophthalmology, Medical University of Lublin, Chmielna 1, 20-079 Lublin, Poland

**Keywords:** porphyrin, human colon tumor and normal cell lines, PDT, cell death, cytokines

## Abstract

Standard in vitro analyses determining the activity of different compounds included in the chemotherapy of colon cancer are currently insufficient. New ideas, such as photodynamic therapy (PDT), may bring tangible benefits. The aim of this study was to show that the biological activity of selected free-base and manganese (III) metallated porphyrins differs in the limitation of colon cancer cell growth in vitro. White light irradiation was also hypothesized to initiate a photodynamic effect on tested porphyrins. Manganese porphyrin (>1 μM) significantly decreased the viability of the colon tumor and normal colon epithelial cells, both in light/lack of light conditions, while decreasing a free-base porphyrin after only 3 min of white light irradiation. Both porphyrins interacted with cytostatics in an antagonistic manner. The manganese porphyrin mainly induced apoptosis and necrosis in the tumor, and apoptosis in the normal cells, regardless of light exposure conditions. The free-base porphyrin conducted mainly apoptosis and autophagy. Normal and tumor cells released low levels of IL-1β and IL-10. Tumor cells released a low level of IL-6. Light conditions and porphyrins were influenced at the cytokine level. Tested manganese (III) metallated and free-base porphyrins differ in their activity against human colon cancer cells. The first showed no photodynamic, but a toxic activity, whereas the second expressed high photodynamic action. White light use may induce a photodynamic effect associated with porphyrins.

## 1. Introduction

Cancer is one of the most serious disorders worldwide, mainly due to late detection of the disease being at the metastatic stage or the use of ineffective therapy. For years, traditional surgery, chemotherapy or radiotherapy have been used to treat many types of cancer, including colon carcinoma among others [1]. Currently, in addition to those mentioned, new therapeutic concepts have been introduced. Their task is not only effective treatment, but also limited systemic toxicity or even targeted action. Therefore, new therapies and new therapeutic agents should not only be aimed at effectiveness, but also simultaneously, without reduced resistance or the inducement of allergic effects.

In colorectal carcinoma (CRC) treatment, surgery, chemo-radiotherapy or targeted antibody therapies are currently used [2]. However, photodynamic therapy (PDT) was also found to have beneficial effects against this cancer [3]. PDT was found to protect against recurrence in the pelvis, after colon carcinoma mass resection, and can be suggested to be used for patients with promising and curative hepatic resections [4,5]. The search for new therapeutic methods of CRC, including PDT, has become a necessity, due to the frequently occurring resistance of cancer cells towards chemotherapeutic drugs or radiotherapy, as well as the appearance of non-specific and systemic toxicity in healthy tissues [6]. Based on this premise, the selection of photodynamic therapy seems to be a choice which is moving in the right direction. This method is based on a local, ablative intervention, with the short action of the cytotoxic species; thus, it is actually a minimally invasive procedure [3]. In principle, PDT operation is based on the functionality of the photosensitizer accumulated in pathological tissues, which is activated by an appropriate wavelength of light. The photosensitizer is then aroused, reacts with endogenous oxygen and generates a singlet of oxygen or a reactive oxygen species (ROS), which causes cell death through further oxidation. ROS react with diffusion limited kinetics with a range of biochemical structures in cells such as lipids, aromatic amino acids, the heterocyclic bases, the backbone of nucleic acids, and flavonoids to induce oxidative damage to the cell, thereby causing cell death via apoptosis or necrosis [7]. Porphyrins are the most common and efficient photosensitizers used in PDT because of their absorption in the visible range of the electromagnetic spectrum, long-lived triplet excited state, and efficient phototoxicity toward cancer cells [8]. It is believed that these compounds selectively bind to and accumulate in tumor cells, thereby protecting normal tissue against a phototoxic effect. In the desired active concentrations they are water-soluble, and very importantly, can be removed from the body within a reasonable time, thus preventing photosensitive reaction development [9]. Different types of porphyrins and metalloporphyrins have also been investigated for biomedical imaging applications, fluorescence tracking, and biosensors [10,11,12]. The advantage of this method is the protection of connective tissue within the radiated area and the possibility of topical or systemic application [3,13,14]. This is especially important for the curation of tissues difficult to reach and growing tumors exophytically, for example. An interesting potential example of PDT applications could be the curation of primary and metastatic CRC. This could be considered as a first-line treatment, as well as a palliative option [3,15,16,17]. Moreover, photodynamic treatments of CRC may be combined with traditional (chemo- or radiotherapy) curative methods or immunotherapy to obtain better therapeutic effects, with a simultaneous reduction in the concentrations of applied drug doses [6,18]. On the other hand, PDT at high doses was found to induce local inflammation, leading to intestinal obstruction or rectal dysfunction. This activity, in addition to possible cutaneous phototoxicity, could be considered as an adverse side effect [19]. However, this therapeutic approach, inducing local acute inflammation, is closely associated with the involvement of an anticancer adaptive immune response which, in turn, should be considered as a desirable outcome [20]. The main role in this mechanism is played by the CD8+ T cells, while supported by the CD4+ T helper cells [20,21]. Therefore, it can be accepted that photodynamic therapy is intertwined with immunotherapy in a specific anti-tumor activity [22]. Nevertheless, PDT’s biological activity, including potential therapy, depends on the kind of photosensitizer used. Generally, PDT seems to be a reaction with a systemic therapeutic effect rather than a local response activity [23].

Therefore, the main scientific hypothesis of this paper was the assumption that base-free and metal-substituted porphyrins exert a different anticancer effect. In order to verify our hypothesis, differences in the biological activity of free-base and manganese (III) metallated porphyrin were tested in a colon carcinoma cell culture model. Moreover, the assumption that the white light irradiation of porphyrins may induce a photodynamic effect was introduced.

## 2. Materials and Methods

### 2.1. Tested Substances and General

5,10,15,20-Tetraphenyl-21H,23H-porphine manganese(III) chloride (further used in short Mn) was purchased from Sigma-Aldrich Co. LLC, (St. Louis, MO, USA) at a purity of 95%, absorption in water with 1% methanol: λmax 467 nm, λmax 566 nm, λmax 600 nm.

5,10,15,20-Tetrakis(1-methyl-4-pyridinio)porphyrin tetra(p-toluenesulfonate) (further used in short FB) was purchased from Sigma-Aldrich Co. LLC, (St. Louis, MO, USA) at a purity of 97%, absorption in water: λmax 421 nm, λmax 518 nm.

**Figure 1:**
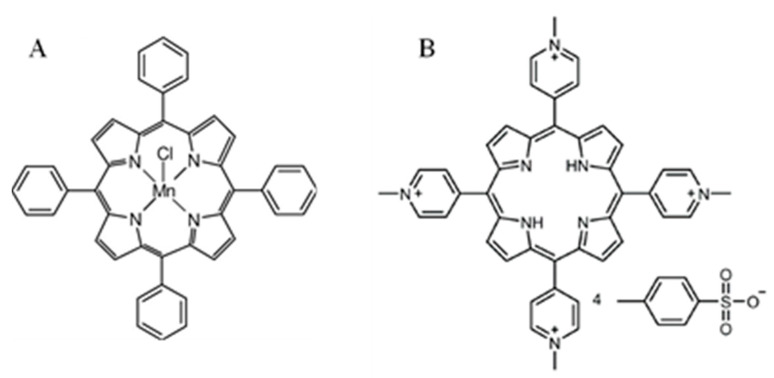
Molecular structures of the porphyrins used in the study; (**A**) 5,10,15,20-tetraphenyl-21H,23H-porphine manganese(III) chloride, and (**B**) 5,10,15,20-tetrakis(1-methyl-4-pyridinio)porphyrin tetra(p-toluenesulfonate).

The molecular structures of the porphyrins used in the study are presented in Figure 1.

Predetermined amounts of the porphyrins were dissolved in dimethyl sulfoxide (DMSO, Sigma-Aldrich Co. LLC, St. Louis, MO, USA) to obtain stock solutions (100 Mm—Mn, 50 mM—FB). Working solutions were prepared by dissolving the stock solutions in the culture medium. A 0.5–100 μM range of concentrations was used in the experiments. During the incubation of cells with the porphyrins, culture plates were sealed with aluminum foil. Experiments were conducted in two separate conditions: a lack of light (for the duration of the experiment) and a short exposure to white light (3 min). Six fluorescent lamps (Osram Lumilux Cool White, Wilmington, MA, USA, with a total power of 90 W) were used as the source of visible light, in accordance with the method described by Lipke et al. [24] and Buczek et al. [25]. Each well of the culture plates was irradiated with a vertical array 20 cm from the plate. The emission spectrum of the visible fluorescent lamps is provided in the supplementary materials. The irradiance of light was measured using a LaserCheck power meter (Coherent, Wilsonville, OR, USA); these were 6 mW/cm^2^, 2.36 mW/cm^2^, 2.20 mW/cm^2^ and 1.68 mW/cm^2^ at 421 nm, 467 nm, 518 nm and 566 nm, respectively. The photon flux density (120 µmol/m^2^s) was measured with a fito-photometer sensor (Optel FR20, Opole, Poland).

Neutral red, 3-(4,5-dimethylthiazole-2-yl)-2,5-diphenyltetrazolium bromide (MTT), 5-fluorouracil, leucovorin, camptothecin, propidium iodide, Hoechst 33342, acridine orange and lipopolysaccharide (LPS) from Escherichia coli, serotype 0111:B4, were obtained from Sigma-Aldrich Co. LLC, (St. Louis, MO, USA). ELISA kits for tests were purchased from BD Biosciences (San Jose, CA, USA) and Roche (Welwyn Garden City, UK).

### 2.2. Cell Cultures

An HT29 cell line (ATCC^®^ No. HTB–38™), human colorectal adenocarcinoma (grade I), was cultured in RPMI 1640 medium, supplemented with 10% fetal calf serum (FCS) (GibcoTM, Paisley, UK) and antibiotics (100 U/mL penicillin, 100 μg/mL streptomycin and 0.25 μg/mL amphotericin B) (GibcoTM, Paisley, UK) at 37 °C, in a humidified atmosphere with 5% CO_2_.

CCD 841 CoTr (ATCC^®^ No. CRL–1807™), human normal colon epithelial cells (SV40 transformed), were cultured in RPMI 1640 + DMEM (1:1) medium (Sigma-Aldrich Co. LLC, St. Louis, MO, USA) supplemented with 10% FCS and antibiotics at 34 °C, in a humidified atmosphere with 5% CO_2_.

### 2.3. Neutral Red (NR) Uptake Assay

The method was based on the ability of living cells to uptake and lysosomal accumulation of the neutral red (dye). Dead or damaged cells cannot take up this substance [26].

Cells were grown for 24 h in 96-well multiplates, in 100 μL of culture medium, with appropriate concentrations of porphyrins. Subsequently, the medium was discarded, and a 0.4% NR solution medium was added to each well. The plate was incubated for 3 h at 37 °C, in a humidified atmosphere with 5% CO_2_. After incubation, the dye-containing medium was removed, and the cells were fixed with 1% CaCl_2_ in 4% paraformaldehyde (200 μL). Afterwards, the incorporated dye was solubilized using 1% acetic acid, in a 50% ethanol solution (100 μL). The plates were gently shaken for 20 min at room temperature, and the absorbance of the extracted dye was measured spectrophotometrically at 540 nm, using a microplate reader (Molecular Devices Corp., Emax, Menlo Park, CA, USA).

### 2.4. MTT Assay

Cell sensitivity to porphyrins was tested in a standard spectrophotometric (MTT) assay, according to the method of Mosmann [27]. The MTT test is based on the conversion of yellow tetrazolium salt by viable cells to purple crystals of formazan (catalyzed by mitochondrial succinic dehydrogenase).

After 24 h of incubation with porphyrins, cells had grown in 96-well multiplates in 100 µL of culture medium and were subsequently incubated for 3 h with a MTT solution (5 mg/mL). Formazan crystals were solubilized overnight in a 10% sodium dodecyl sulfate, in a 0.01 M HCl mixture. The product was quantified spectrophotometrically by absorbance measurements at the 570 nm wavelength, using an Emax microplate reader (Molecular Devices Corp., Menlo Park, CA, USA).

### 2.5. Interactions between Porphyrins and Cytostatics

The interaction between different drugs in combination (synergism, additivity, and antagonism) has often been estimated through the “combination index”, an algorithm according to Paduch et al. [28]:

A = a/X + a/Y(1)

where A is the combination index, X is the cytotoxicity of substance 1, Y is the cytotoxicity of substance 2, and a is the cytotoxicity of substance 1 in combination with substance 2.

If the value is lower than 0.7, the combination of drugs indicates synergism; in the range from 0.7 to 1.3, additivity; higher than 1.3, antagonism.

After 24 h of incubation with porphyrins (1 μM and 5 μM Mn; 10 μM and 100 μM FB), three mixtures of cytostatics (1–20 µM 5-FU and 0.2 µM LV; 2–3 µM CPT11; 3–20 5-FU, 0.2 µM LV and 2.1 µM CPT11) and porphyrins were combined with cytostatics in 96-well multiplates, the medium was discarded and the procedure, with an NR assay, was performed.

### 2.6. Double Time Analysis

Cell doubling time analysis was determined by counting cells in a Thoma chamber, and calculated according to the following formula [29]:

DT = (t−t0)log2/(logN−logN0)(2)

where DT is the time required to double cell population, t is the hours of experiment from t0, t0 is time 0 (start time, 0 h), N0 is the level of cells at t0, and N is the level of cells after t.

Cells were grown for time “t” in 6-well multiplates, in a culture medium with appropriate concentrations of porphyrins. Cells were counted at time “t0” (N0) and after time “t” (N). According to the formula, the time (h)required for cells to double in the presence of porphyrins was calculated.

### 2.7. Staining of Apoptosis, Necrosis and Autophagy

This method allows for the identification of dead cells, based upon the detection of fluorescence dyes bonded to DNA (propidium iodide, Hoechst 33342, acridine orange) [30,31].

#### 2.7.1. Apoptosis and Necrosis

After 24 h of cell incubation with porphyrins (1 µM, 5 µM, 10 µM Mn; 1 µM, 10 µM and 100 µM FB), cells were suspended in Hoechst 33342 (0.24 mg/mL) and a propidium iodide (0.15 mg/mL) solution and were incubated at 37 °C, in CO_2_, in the dark, for 5 min.

#### 2.7.2. Autophagy

After 24 h of cells incubation with porphyrins (1 µM, 5 µM, 10 µM Mn; 1 µM, 10 µM and 100 µM FB), cells were suspended in acridine orange (5 μg/mL) and were incubated at 37 °C, in CO_2_, in the dark for 15 min.

Assessment of the type of cell death (apoptosis, necrosis, autophagy) was conducted using a confocal microscope; Zeiss Axiovert 200 M CLSM (Zeiss) at 100× magnification. Image analysis was performed using analysis Image Processing (Carl Zeiss AG, Oberkochen, Germany) (percentage of dead cells).

### 2.8. Cell Death Detection ELISAPLUS Assay

The levels of human mono- and oligonucleosomes were tested immunoenzymatically (Cell Death Detection ELISA) in a cell culture, using a commercially available kit, according to the manufacturer’s instructions. The optical density of each ELISA sample was determined using a microplate reader, set to 490 nm. Concentrations of nucleosomes were calculated on the basis of a negative and positive control; mono- and oligonucleosomes from 5 × 10^2^ cells/mL were the detection limits. The concentration of porphyrins was analogical to cell death staining.

### 2.9. ELISA Assay

The levels of human IL-1β, IL-6 and IL-10 were tested immunoenzymatically (ELISA) in culture supernatants, using commercially available kits, according to the manufacturer’s instructions. The concentrations of porphyrins used in the study were as follows: 0.1 μM; 0.5 μM, 1 μM Mn; 0.1 μM; 1 μM and 5 μM FB. The optical density of each ELISA sample was determined using a microplate reader, set to 450 nm, with a correction wavelength of 570 nm. Interleukin concentrations were calculated on the basis of a standard curve. The cytokine detection limits were: 0.8 pg/mL (IL-1β), 2.2 pg/mL (IL-6) and 2 pg/mL (IL-10).

In parallel experiments, cells were pre-activated for 2 h with 10 μg/mL LPS from Escherichia coli, serotype 0111:B4, and then incubated with porphyrins for 24 h.

### 2.10. Statistics

The results are presented as the means ± SD of three independent experiments (*n* = 3). The data were analyzed using one-way analysis of variance (ANOVA), followed by Bonferroni’s multiple comparison post hoc test in a GraphPad Prism 5.0 program (GraphPad Software, San Diego, CA, USA). Differences of *p* ≤ 0.05 were considered significant.

## 3. Results

### 3.1. Cytotoxicity of Porphyrins

To examine the cytotoxicity of porphyrins, two parallel experiments were performed—NR uptake and MTT assay.

**Figure 2:**
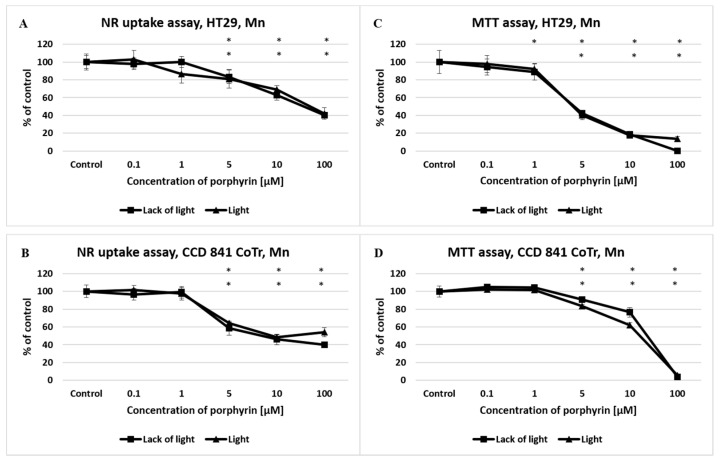
The effect of manganese porphyrin (Mn) on viability and mitochondrial metabolism of the HT29 cell line (**A**,**C**) and CCD 841 CoTr cells (**B**,**D**) after light induction and the absence of light. The neutral red (NR) uptake assay (**A**,**B**) and the MTT test (**C**,**D**). The results are presented as a percentage of the controls, arbitrarily set to 100%. The figures show averages of three independent experiments. *—Statistical significance was analyzed at *p* ≤ 0.05.

A significant decrease in the viability of colorectal and normal cells was observed when the manganese porphyrin (Mn) was used at a concentration of 1 μM, in both tests (NR and MTT assay) and in both conditions (light/lack of light) (Figure 2). No significant differences were observed in results obtained in different light conditions, as compared with the control.

**Figure 3:**
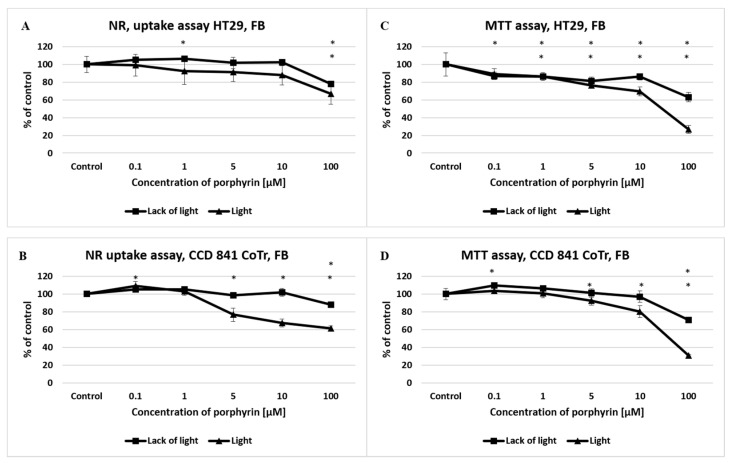
The effect of free-base porphyrin (FB) on viability and mitochondrial metabolism of the HT29 cell line (**A**,**C**) and CCD 841 CoTr cells (**B**,**D**) after light induction and the absence of light. The neutral red (NR) uptake assay (**A**,**B**) and the MTT test (**C**,**D**). The results are presented as a percentage of the controls, arbitrarily set to 100%. The figures show averages of three independent experiments. *—Statistical significance was analyzed at *p* ≤ 0.05.

In the case of free-base porphyrin (FB), no significant differences were observed in cell integrity (NR uptake) and mitochondrial metabolism (MTT) in the of lack of light condition. In the presence of white light, there was a significant decrease in the viability of both cell lines (from concentrations 1 μM, in both tests) in comparison to the control (Figure 3).

MTT assay revealed higher cytotoxicity to the cells of both lines. The half-maximal effective concentration (EC50) values in the tested range of concentrations are presented in Table 1.

### 3.2. Interactions between Porphyrins and Cytostatics

In almost all conditions, for both porphyrins, antagonism of their interactions with cytostatics was observed. Only an additive impact at a concentration of 5 μM of manganese porphyrin with mixture number 3 (20 µM 5-FU with 2.1 µM CPT-11) for the CCD 841 CoTr cells (in both light conditions) was observed.

To test the effect of porphyrins on cell proliferation, double time analysis was used. The study demonstrated the complete inhibition of cell proliferation after exceeding a concentration of 1 μM (Mn, FB) for both cell lines. There were no significant differences in the acquired time to cell proliferation between light conditions and the presence of manganese porphyrin (HT29, CCD 841 CoTr). In the case of free-base porphyrin (FB), significant differences in cell proliferation in light conditions were found. In the presence of light, cell proliferation was slower, starting from the lowest concentration (0.1 μM). The difference between both conditions was 25.9 h for HT29 cell line and 25.4 h for CCD 841 CoTr cells (Table 2 and Table 3).

### 3.3. Cell Death Mode Analysis

**Figure 4:**
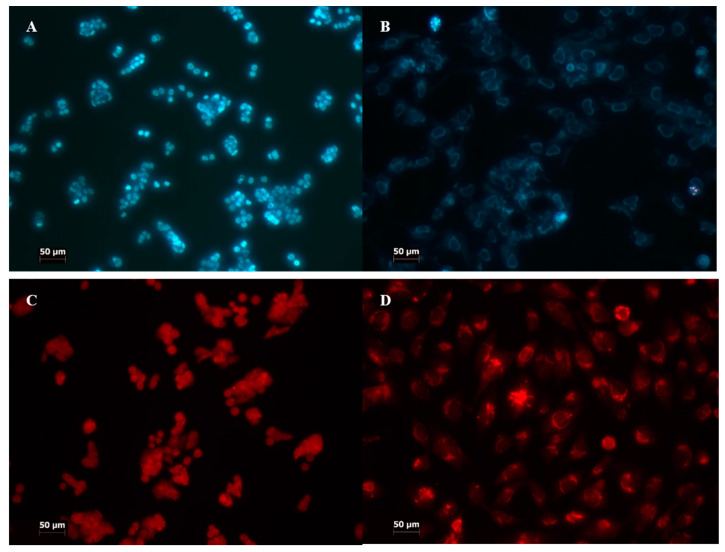
Staining for different kinds of cell death analysis. (**A**,**B**) staining with propidium iodide and Hoechst 33342; (**C**,**D**) staining with acridine orange; (**A**,**C**) HT29; (**B**,**D**) CCD 841 CoTr. The fluorescence was excited at the wavelength λ = 420 nm. Cells were treated with porphyrins at a concentration of 10 µM and exposed to white light irradiation for 3 min. The cells stained blue were identified as living, whereas a significant intensity of fluorescence with the presence of nuclear graininess confirmed apoptosis induction. Cells stained red were assumed to be dead. Magnification 100×. Bar = 50 µm.

To determine the kind of cell death, several fluorescence stainings were performed (apoptosis, necrosis, and autophagy) (Figure 4).

In the case of manganese porphyrin, there were higher levels of apoptosis and necrosis (3–5%) with the use of the 10 μM concentration at the HT29 cell line, independent of light/non-light conditions. A high level of apoptosis (10%) was observed in CCD 841 CoTr cells, when a 10 μM concentration of Mn porphyrin was used. There were also no significant differences between different light conditions. Free-base porphyrin conducted a low level of apoptosis and autophagy. There was no more than 1% at the HT29 cell line and 2% at CCD 841 CoTr, regardless of light conditions.

**Figure 5:**
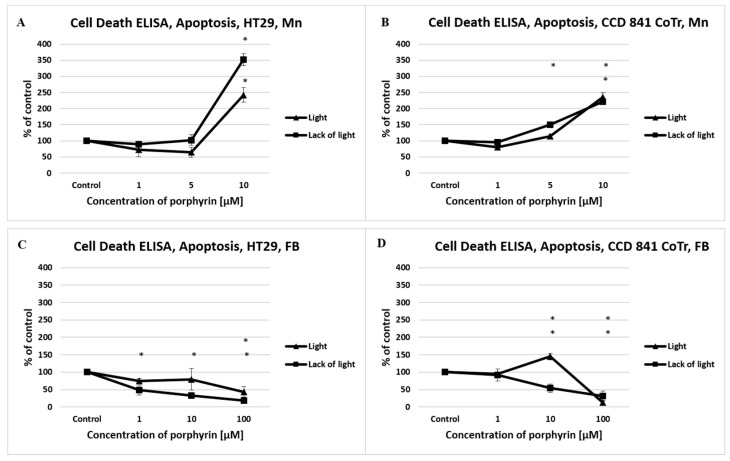
The level of apoptosis induced by manganese porphyrin (Mn) and free-base porphyrin (FB) analyzed by a Cell Death Detection ELISAPLUS assay. Columns and bars show the mean standard deviation (*n* = 3). *—Statistical significance was analyzed at *p* < 0.05.

In view of the results obtained from the fluorescence staining, apoptosis was measured by the Cell Death Detection ELISA kit. A strong proapoptotic effect of manganese porphyrin to the HT29 cell line was shown as reaching 351% (lack of light) and 242% (light), and to CCD 841 CoTr cells, reaching 221% (lack of light) and 234% (light) relative to the control. Free-base porphyrin showed a much weaker propoptotic activity, even at the highest concentration of porphyrin applied (Figure 5).

### 3.4. Inflammation and Tumor Microenvironment

In order to verify the level of human interleukin IL-1β, IL-6, IL-10 in post-culture supernatants, ELISAs were conducted.

**Figure 6:**
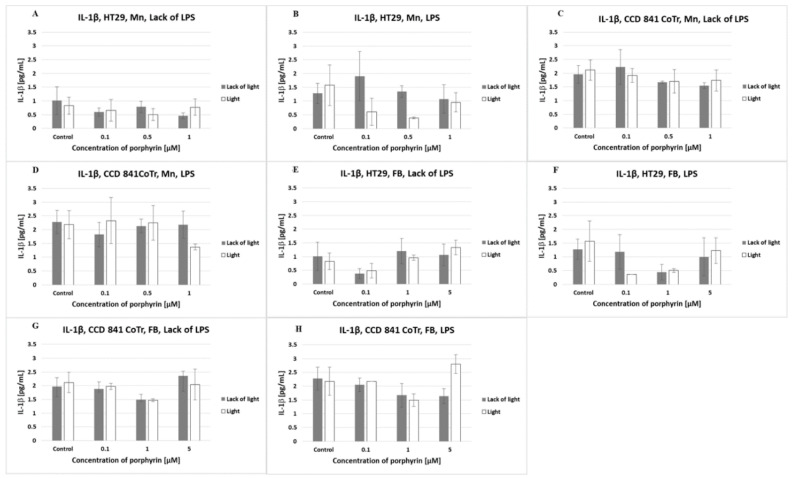
IL-1β secretion at the HT29 cell line (**A**,**B**,**E**,**F**) and culture of CCD 841 CoTr cells (**C**,**D**,**G**,**H**) over 24 h of incubation with manganese (Mn) (**A**–**D**) and free-base (FB) (**E**–**H**) porphyrins. ELISA test. Cells treated with porphyrin compared with a non-treated control culture. Cells treated with porphyrin compared with cells treated with porphyrin after pre-incubation with LPS. Columns and bars show the mean ± standard deviation (*n* = 3).

There was a very low level of IL-1β produced by normal and tumor cells. At the level of interleukin, the analyzed conditions (light/lack of light; Mn/FB, LPS) did not have any effect (Figure 6).

**Figure 7:**
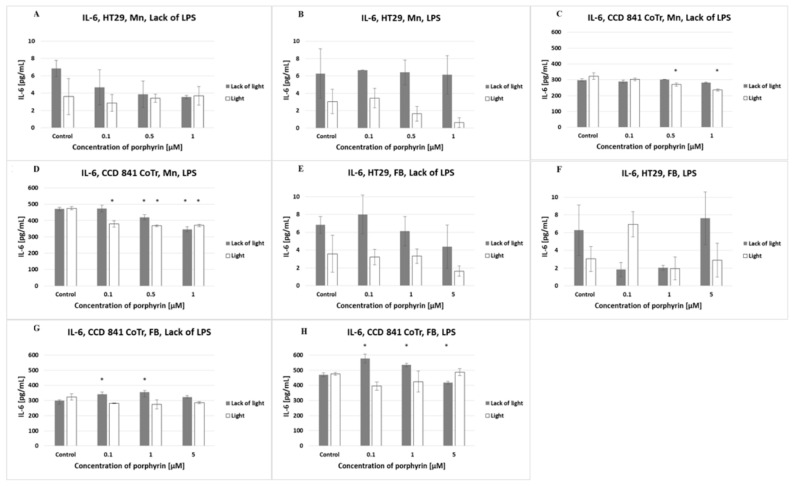
IL-6 secretion at the HT29 cell line (**A**,**B**,**E**,**F**) and culture of CCD 841 CoTr cells (**C**,**D**,**G**,**H**) over 24 h of incubation with manganese (Mn) (**A**–**D**) and free-base (FB) (**E**–**H**) porphyrins. ELISA test. Cells treated with porphyrin compared with a non-treated control culture. Cells treated with porphyrin compared with cells treated with porphyrin after pre-incubation with LPS. Columns and bars show the mean ± standard deviation (*n* = 3). *—Statistical significance was analyzed at *p* < 0.05.

The HT29 cell line released a low level of IL-6 (lower than 10 pg/mL), in contrast to CCD 841 CoTr, which produced a very high level of this cytokine (above 300 pg/mL). Pre-incubation of cells for 2 h with LPS significantly increased the level of IL-6 produced by normal cells (above 500 pg/mL). Tumor cell stimulation by LPS had no significant effect on the increase in the IL-6 level (Figure 7).

The amount of IL-6 released by HT29 cells decreased after the addition of increasing concentrations of manganese porphyrin in a lack of LPS and lack of light. In a lack of LPS and in the presence of light, this porphyrin did not affect the release of IL-6 by HT29 cells. Pre-incubation of tumor cells with LPS induced lower IL-6 release by irradiated cells as compared with non-irradiated cells. Analyzing the level of IL-6 released by normal cells (CCD 841 CoTr), it was found that increasing concentrations of manganese porphyrin (0.5 and 1 µM) reduced the amount of cytokine released under irradiation and in a lack of LPS. As the concentration of manganese porphyrin increased, and irrespective of the irradiation or lack of it, a decrease in the amount of IL-6 released by normal cells that were pre-incubated with LPS was found. Free-base porphyrin only had a slight effect on the amount of IL-6 in all tested conditions.

**Figure 8:**
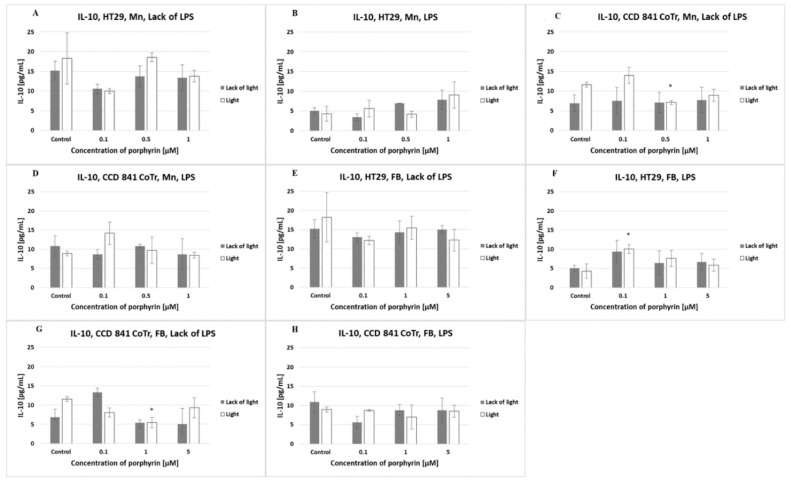
IL-10 secretion at the HT29 cell line (**A**,**B**,**E**,**F**) and culture of CCD 841 CoTr cells (**C**,**D**,**G**,**H**) over 24 h of incubation with manganese (Mn) (**A**–**D**) and free-base (FB) (**E**–**H**) porphyrins. ELISA test. Cells treated with porphyrin compared with a non-treated control culture. Cells treated with porphyrin compared with cells treated with porphyrin after pre-incubation with LPS. Columns and bars show the mean ± standard deviation (*n* = 3). *—Statistical significance was analyzed at *p* < 0.05.

The amount of IL-10 produced by cells of both lines was lower than 20 pg/mL (regardless of the different conditions). No significant differences were observed between the porphyrins or light conditions used (Figure 8).

## 4. Discussion

For many years, classic methods of cancer healing included mainly chemotherapy, radiotherapy and surgery. These therapeutic procedures, in many cases, were effective, but the application of radiation or cytostatic agents entailed some side effects, life-threatening complications, and fairly quickly induced tumor cell resistance. For these reasons, there was a need to introduce some new therapeutic agents and cancer treatment methods to overcome such problems. The solution was the application of targeted-based cancer therapies (TBCTs) to a large extent, but under certain conditions, comprising photodynamic therapy (PDT) [1]. This is a minimally invasive therapeutic method, with high local efficiency [32]. It may be applied systemically, but also topically, mainly because of the advantages of many substances used as photosensitizers, including porphyrins. In the context of selective impact of porphyrins on different cells, it was also shown that manganese porphyrins may serve as potent radioprotectors, shielding normal tissues but not tumors from radiation. Kosmacek et al. [33] found that MnTnBuOE-2-PyP manganese porphyrin inhibited morphological changes in normal fibroblasts, enabling them to maintain their proper, healthy cellular phenotype after radiation exposure. On the other hand, they found that this porphyrin does not protect colorectal cells exposed to the same stress conditions. Moreover, the combination of MnTnBuOE-2-PyP manganese porphyrin with Mitomycin C or similar to our study, with 5-fluorouracil, enhanced the cytostatics’ ability to reduce the proliferative activity of human colon tumor cells [33]. This was a synergistic effect, mainly inducing apoptotic or necrotic tumor cell death [33,34]. MnTE-2-PyP, in turn, reduced epithelial–mesenchymal transition (EMT), MMP production, and thus limited tumor cell migration/invasion in TGF-β stimulated colorectal cancer [35,36]. Radiation, chemoradiation or the photodynamic activity of some chemical compounds are strong stressors for cells. These therapeutic methods or applied compounds exert a strong impact on cell morphology, as well as on molecular pathways responsible for cell growth and viability. Manganese porphyrins down-regulated pathways of HIF-1β, NF-κB, TGF-β and VEGF-A, and decreased P53 protein levels. Changes in these mechanisms, after the porphyrin impact of stress, may prevent senescence in normal, but not tumor colorectal cells [33]. Similar observations have been made in our study, indicating a different, dual effect of manganese and base-free porphyrins on the features of tumor and normal cells.

The mechanism of porphyrin action is generally based on reactive oxygen species (ROS) formation, including O_2_-, H_2_O_2_, and OH^.^, and the most important therapeutically, ^1^O_2_, upon activation by light. One of the most important sources of ROS in cells’ microenvironment, after irradiation, is NADPH oxidase 4 (NOX 4). However, manganese porphyrin MnTnBuOE-2-PyP reduces NOX 4 ROS production by inhibition of the p38 MAPK-Akt pathway or NF-κB, and thus protects normal cells from radiation-induced damage. This confirms a therapeutically preferred dual role of Mn porphyrins for use as a stress factor for cancer treatment [26]. ROS strongly interact with cellular membranes, oxidizing amino acids, fatty acids and cholesterol, and interact with double-stranded DNA (dsDNA) cleaving it or interacting with protein synthesis [37,38,39,40]. A consequence of these interactions is apoptotic cell death.

Porphyrins may be used for the treatment of many cancers, but those which can be visualized by endoscopy have received special attention. This is because of the possibility of reductions in the surgical operation scope or even avoiding surgeries, when endoscopic photodynamic therapy (EPDT) would be used. Such treatment appeared to be safe and beneficial to healing colorectal cancer (CRC), but due to the lack of randomized trials, it is not applied in practice [19].

Our in vitro study indicates the potential of porphyrins in the treatment of colon carcinoma cells. We compared the biological activity of free-base and manganese (III) metallated porphyrin and found significant differences.

Manganese (III) metallated porphyrin showed a predominantly typical cytotoxic activity, whereas free-base porphyrin expressed a photodynamic action after irradiation with white light. This is in accordance with observations indicating that the lack of coordinated metal ions in porphyrins makes them more efficient in PDT than metallophotosensitizers. However, the presence of coordinated metal ion does not preclude such porphyrins from their use in clinical purposes. This is associated with their specific properties, such as stability or good solubility [37]. It was also revealed that Mn porphyrins may radiosensitize different tumors to radiotherapy, as well as protect normal tissue during such therapy. Mn porphyrins have also been shown to limit brain-damaging effects or prevent neuropathic pain during chemotherapy [41]. PDT is gaining increasing confidence within clinical treatment. However, the endoscopic photodynamic system is still very poorly developed for in vivo tests [19]. Therefore, our tests determined whether white light, which is commonly used in endoscopic visualization, may be useful to initiate a photodynamic reaction with porphyrins. Another reason for white light usage was the assumption that porphyrins, irradiated with the whole spectrum of visible light, do not cause significant tissue damage, primarily by necrosis, as is observed after specific wavelength application [42]. It has been reported that porphyrins in other biological systems can be excited by white light, activating/yielding different photochemical reactions with an efficiency not significantly dependent on particular wavelengths of visible light [43,44,45]. As we observed, after porphyrin irradiation with the entire spectrum of visible light, cells died mainly from apoptosis. This is consistent with other studies, indicating that apoptosis is one of the most important kinds of tumor cell death, after PDT application [34,46,47,48]. The mechanism of action is based on ROS release, increased Ca^2+^ concentration and consequently cytochrome c release, downregulation of Bcl-2, upregulation of Bax, as well as caspases or PARP [46,49,50]. It was also found that the Bcl-2 level may be dependent on PDT–photosensitizer concentrations [8]. Apoptosis, however, is not the only mechanism of tumor cell death induced by PDT. The activation of stress kinases is associated with PDT-induced apoptotic or necrotic modes of cell killing. However, the apoptotic pathway of cell death inducement after PDT is strictly associated with a proper photosensitizer dose and structure, as well as the cellular component which, depending on the tissue of origin, expresses a different sensitivity to stress impacts. It was shown that inhibition of the JNK and p38 MAPK, but not ERK pathways, specifically enhanced apoptotic cell death [34]. Necrosis may also take place as a dominating cell death mode, depending on the specific photosensitizer used and the kind of tumor cells at which the PDT is aimed [6,32]. On the other hand, autophagy may exert a potential cytoprotective effect against PDT in colon carcinomas. Moreover, it was found that the inhibition of autophagy may potentiate the proapoptotic effect of porphyrin IX (PpIX)-mediated PDT [23]. Therefore, targeting this process may improve the sensitivity of tumor cells to photodamage [6,13]. The limited sensitivity of colon tumor cells to porphyrins may not necessarily be a result of white light application, but mainly drug resistance inducement by ATP-binding cassette subfamily G2 (ABCG2) proteins [51,52]. In our study, we also revealed that porphyrins and cytostatics, added simultaneously, exert an antagonistic effect of interactions. This may be an effect of the interferential action of both compounds, influencing similar targets in tumor cells. Moreover, it has already been revealed that porphyrins exhibit significantly greater anti-tumor potency than that observed in classic chemotherapeutic drugs [49]. Therefore, the diminished activity of porphyrin and cytostatic drug added together may represent either localization of their activity at the same cell-death pathways or a dominant position of PDT over classic chemotherapy, associated with the displacement of drug activity by porphyrin effects.

We found (data not shown) that porphyrins are initially localized in the outer region of the cytoplasm, but after the incubation time is changed, this compound was found around the nucleus. In general, intracellular localization of photosensitizers is very important for their photodynamic action. This depends on many factors, such as the degree of aggregation, solubility, and photosensitizer charge [13]. Porphyrins accumulate intracellularly in endocytic vesicles, and are released into the cytosol after light exposure [30]. In this cellular compartment, porphyrins accumulate in crucial targeted sites, including mitochondrial or nucleus membranes and plasma membranes [13,41,53]. Our study confirmed these observations. It could also be speculated that the permeability of mitochondrial membranes due to PDT action, for example, and most likely associated with lysosomal damage, may result in a direct signal for tumor cells to enter the apoptosis pathway.

In addition to the direct cytotoxic effect of porphyrins on tumor cells, equally important is the process of changing local immune responses by these compounds. We found that specific changes affected the level of IL-6 and COX-2. This indicates that porphyrins have a special impact on the inflammatory status of treated tissues. This is consistent with other tests, indicating that IL-6 expression was increased in the sera of mice after PDT application [54,55]. IL-6 is a proinflammatory cytokine that influences acute-phase protein release, among others COX-2. Such tumor microenvironmental changes, after porphyrin addition, may modulate and promote adaptive immune responses, which, consequently, may reduce tumor growth. The inducement of antigen-specific antitumor immunity, as well as the reorientation of immune responses, may finally be beneficial, from the standpoint of high clinical effectiveness.

## 5. Conclusions

In conclusion, we found that the biological activity of free-base and manganese (III) metallated porphyrins differs in their activity on human colon tumor cells in vitro. Metalled porphyrin expresses general toxicity, whereas free-base porphyrin expresses it only after light exposure. This suggests that the potential PDT therapy of colon carcinoma could bring greater benefits when free-base porphyrin is used as a photosensitizer. Furthermore, we showed that it is not necessary to obligatorily use selected wavelength radiation, but simply white light, commonly adapted to endoscopic colonoscopy. We suppose that porphyrin irradiation with white light may induce a mild pro-apoptotic effect to colon tumor cells and the tumor microenvironment, and could thus be advantageous over classic chemotherapy methods, when it is actually desirable to only reduce tumor growth as neoadjuvant treatment, for example, with limited side effects for the whole organism.

## Figures and Tables

**Table 1:** EC50 values obtained after 24 h incubation of human colon tumor cell (HT29) and human normal colonic epithelial cell (CCD 841 CoTr) with manganese porphyrin (Mn) and free-base porphyrin (FB) after light induction and in the absence of light (MTT and neutral red analysis). Statistical significance was analyzed at *p* < 0.05.

		HT29		CCD 841 CoTr	
		Lack of Light	Light	Lack of Light	Light
NR	Mn	44 µM	55.5 µM	11.3 µM	8.6 µM
	FB	nd *	nd *	nd *	nd *
MTT	Mn	3.2 µM	3.5 µM	18 µM	14.6 µM
	FB	nd *	31.2 µM	nd *	42.6 µM

* nd, not detectable EC50 value in the tested range.

**Table 2:** Time required to double the population of the HT29 cell line (h) in different conditions of incubation: porphyrin (Mn, FB), their concentration and light induction/light absence. The doubling time (DT) for HT29 cells used as a control was 19.6 h. Statistical significance was analyzed at *p* < 0.05.

	Mn	Mn + Light	FB	FB + Light
0.1 μM	51.7 ± 23.7	53.6 ± 18.1	35.7 ± 3.9	61.6 ± 16.4
1 μM	147.5 ± 56.8	nd	43.9 ± 2.9	90.5 ± 74.2
5 μM	nd	nd	nd	nd
10 μM	nd	nd	nd	nd
100 μM	nd	nd	nd	nd

nd, not detectable value in the tested range.

**Table 3:** Time required to double the population of the CCD 841 CoTr cells (h) in different conditions of incubation: porphyrin (Mn, FB), their concentration and light induction/light absence. The doubling time (DT) for CCD 841 CoTr cells used as a control was 30.1 h. Statistical significance was analyzed at *p* < 0.05.

	Mn	Mn + Light	FB	FB + Light
0.1 μM	58.4 ± 25.0	56.9 ± 21.9	69.5 ± 29.1	94.9 ± 36.3
1 μM	116.3 ± 44.7	nd	114.2 ± 34.6	nd
5 μM	nd	nd	nd	nd
10 μM	nd	nd	nd	nd
100 μM	nd	nd	nd	nd

nd, not detectable value in the tested range.

## Data Availability

Data are available from the authors.

## Supplementary Materials

The following supporting information can be downloaded at: https://www.mdpi.com/article/10.3390/molecules27062006/s1, Figure S1: Emission spectrum of visible fluorescent lamps.

## Sample Availability

Samples of the compounds are available from the authors.
