# Supplementary material for: Assessing the In Vitro Activity of Selected Porphyrins in Human Colorectal Cancer Cells
Source: Molecules. 2022 Mar 21;27(6):2006. doi: 10.3390/molecules27062006 (PMC8955395; doi:10.3390/molecules27062006)

Supplementary materials

Emission spectrum of visible fluorescent lamps (OSRAM L 15W/840, LUMILUX Cool White) used in the experiments.

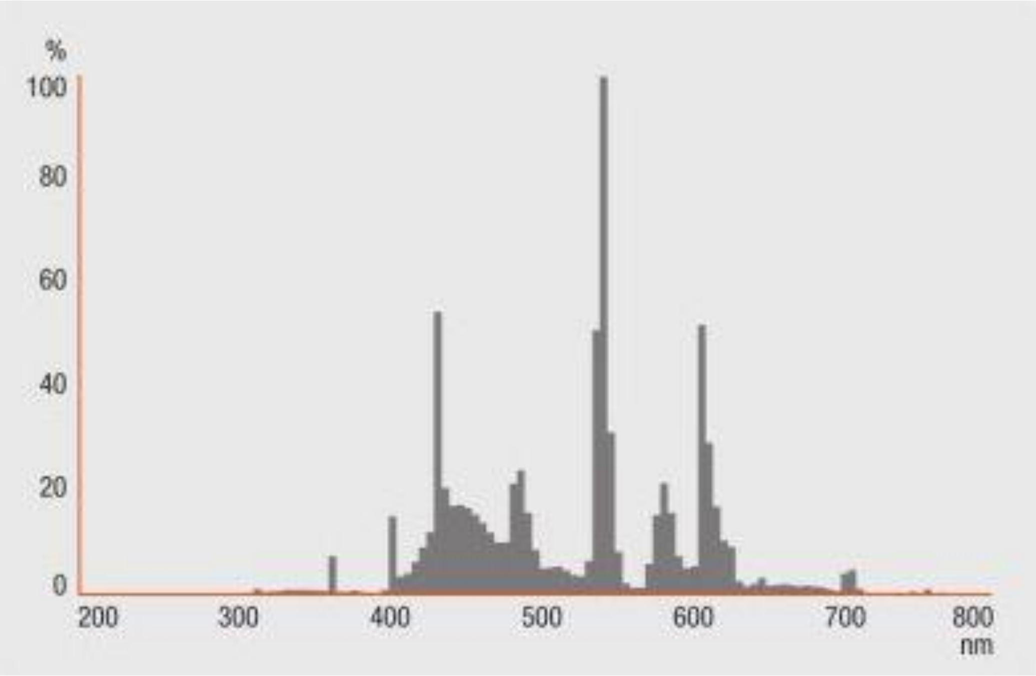

Supplement: Supplementary file 1 [file molecules-27-02006-s001.zip › molecules-1602984-supplementary.pdf]
